# Cell-specific RNA purification to study translatomes of mouse central nervous system

**DOI:** 10.1016/j.xpro.2022.101397

**Published:** 2022-05-20

**Authors:** Isabel Bravo-Ferrer, Baljit S. Khakh, Blanca Díaz-Castro

**Affiliations:** 1 UK Dementia Research Institute, University of Edinburgh, Chancellor’s Building, Edinburgh EH16 4SB, Scotland, UK; 2 Centre for Discovery Brain Sciences, University of Edinburgh, Chancellor’s Building, Edinburgh EH16 4SB, Scotland, UK; 3 Department of Physiology, David Geffen School of Medicine, University of California Los Angeles, Los Angeles, CA 90095-1751, USA; 4 Department of Neurobiology, David Geffen School of Medicine, University of California Los Angeles, Los Angeles, CA 90095-1751, USA

**Keywords:** Cell Biology, Microscopy, Model Organisms, Molecular Biology, Gene Expression, Neuroscience, Systems biology

## Abstract

Cell-specific RNA sequencing has revolutionized the study of cell biology. Here, we present a protocol to assess cell-specific translatomes of genetically targeted cell types. We focus on astrocytes and describe RNA purification using RiboTag tools. Unlike single-cell RNA sequencing, this approach allows high sequencing depth to detect low expression genes, and the exploration of RNAs translated in subcellular compartments. Furthermore, it avoids underestimation of transcripts from cells susceptible to cell isolation procedures. The protocol can be applied to a variety of cell types.

For complete details on the use and execution of this protocol, please refer to Chai et al. (2017), Díaz-Castro et al. (2021), Díaz-Castro et al. (2019), Srinivasan et al. (2016), and Yu et al. (2018).

## Highlights

• A protocol for investigating cell-specific translatomes in mouse brain cells
• Purification of RNA from genetically targeted cells using RiboTag tools
• Recommended antibodies and oligonucleotides to assess brain-cell specificity
• Sample quality assessment tips for generating the most reliable data

## Graphical abstract

**Figure:**
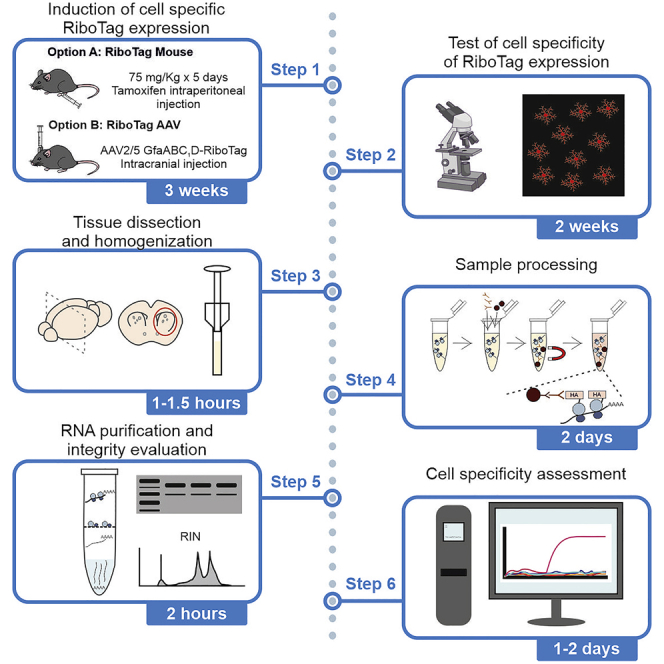

## Before you begin

This protocol describes how to purify RNA from specific cells using RiboTag tools. RiboTag is a technique that consists of expressing Human influenza hemagglutinin (HA) tagged ribosomal subunit RPL22 in a cell-specific manner (Sanz et al., 2009). The expression of this protein will allow the pull down of HA tagged ribosomes with their bound RNAs that can be subsequently purified to perform RNA sequencing (RNA-seq).

### Institutional permissions

Animal experiments were conducted in accordance with the National Institute of Health Guide for the Care and Use of Laboratory Animals and were approved by the Chancellor’s Animal Research Committee at the University of California, Los Angeles. All mice were housed with food and water available ad libitum in a 12 h light/dark environment. All animals were culled during the light cycle, and none were involved in previous studies.

**CRITICAL:** Any experiments on live vertebrates or higher invertebrates must be performed in accordance with relevant institutional and national guidelines and regulations. To perform this protocol, please make sure that you have acquired the permissions from the relevant institutions.

### Induction of cell-specific RiboTag expression

**Timing: 3 weeks**

**Figure 1:**
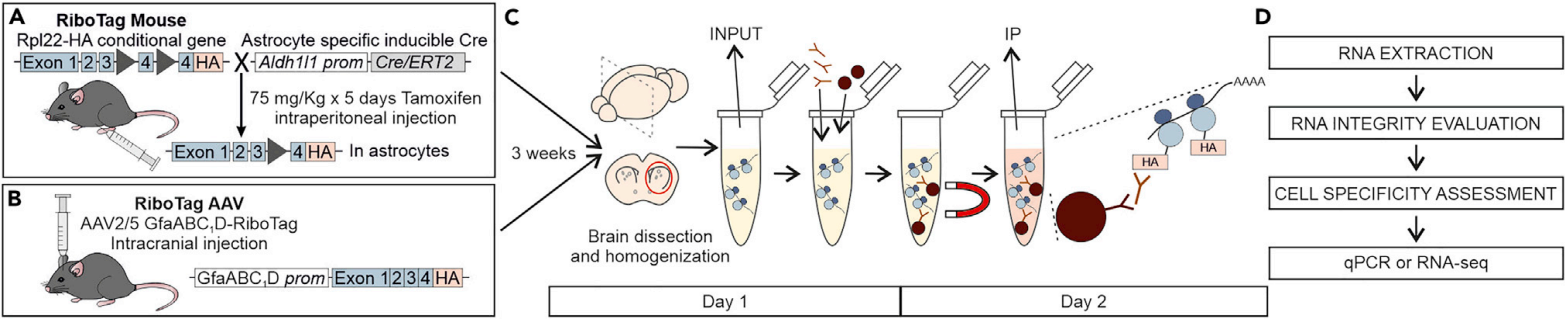
Schematic for the RNA purification protocol (A and B) Representation of RiboTag mouse (A) or RiboTag AAV (B) strategies. (C) Schematic for homogenization and pull-down protocol. (D) Follow up steps.

There are two commonly used ways in which RiboTag expression can be induced in the cell of interest: using mouse models or adeno associated viruses (AAVs) (Figures 1A and 1B).

1. RiboTag mice. This technique was developed by the Amieux lab in 2009 (Sanz et al., 2009). They generated a knock in mouse line that expresses the HA tagged RPL22 ribosomal subunit, under *Rpl22* endogenous promoter, upon Cre recombination. Cell specificity can be controlled by breeding this line to cell-specific Cre expressing mouse lines, e.g., *Aldh1l1-CreERT2* for astrocytes (Srinivasan et al., 2016) (Figure 1A).

***Note:*** When using RiboTag mice crossed with a tamoxifen inducible Cre line, e.g., RiboTag::*Aldh1l1-CreERT2* mice, tamoxifen can be administered intraperitoneally or by gavage at a dose of 75 mg/kg per day for five consecutive days to induce RiboTag expression three weeks before RNA extraction.

2. RiboTag AAVs. In some cases, breeding mouse lines can be inefficient, costly and time consuming, especially when investigating genetic mouse models of disease. To overcome this obstacle, we created astrocyte specific RiboTag expressing AAVs (Díaz-Castro et al., 2019; Yu et al., 2018). These AAVs overexpress RiboTag in astrocytes under a short version of the *Gfap* promoter, *GfaABC*_*1*_*D* (Figure 1B).
  a. To use RiboTag AAVs, microinject the viral particles intracranially in the brain region of interest three weeks before RNA purification.
  b. We recommend injecting approximately 10^10^ genome copies in a total volume of 0.5–0.8 μL.

***Note:*** The AAV RiboTag expression spread will need to be assessed with immunohistochemistry. It may differ between brain regions. If the expression spread is wider than the brain area, it is not a problem as long as you can dissect the area of interest. If not, then lower volumes of AAV may need to be tested. As an example, 10^10^ genome copies in a total volume of 0.8 μL covered approximately 50% of the whole striatum (Díaz-Castro et al., 2019).

The protocol below details the specific steps for purifying astrocyte RNA from mouse striatum. The protocol is described to generate a total of four replicates, each replicate corresponds to the striata of a single mouse. Nonetheless, this protocol can be adjusted to more samples or used to purify RNA from other cells as long as the mouse or AAV tools needed are available. For other applications of RiboTag please see Gao and Zhao (2021) and Sanz et al. (2019).

### Test cell specificity of RiboTag expression

**Timing: 2 weeks**

**Figure 2:**
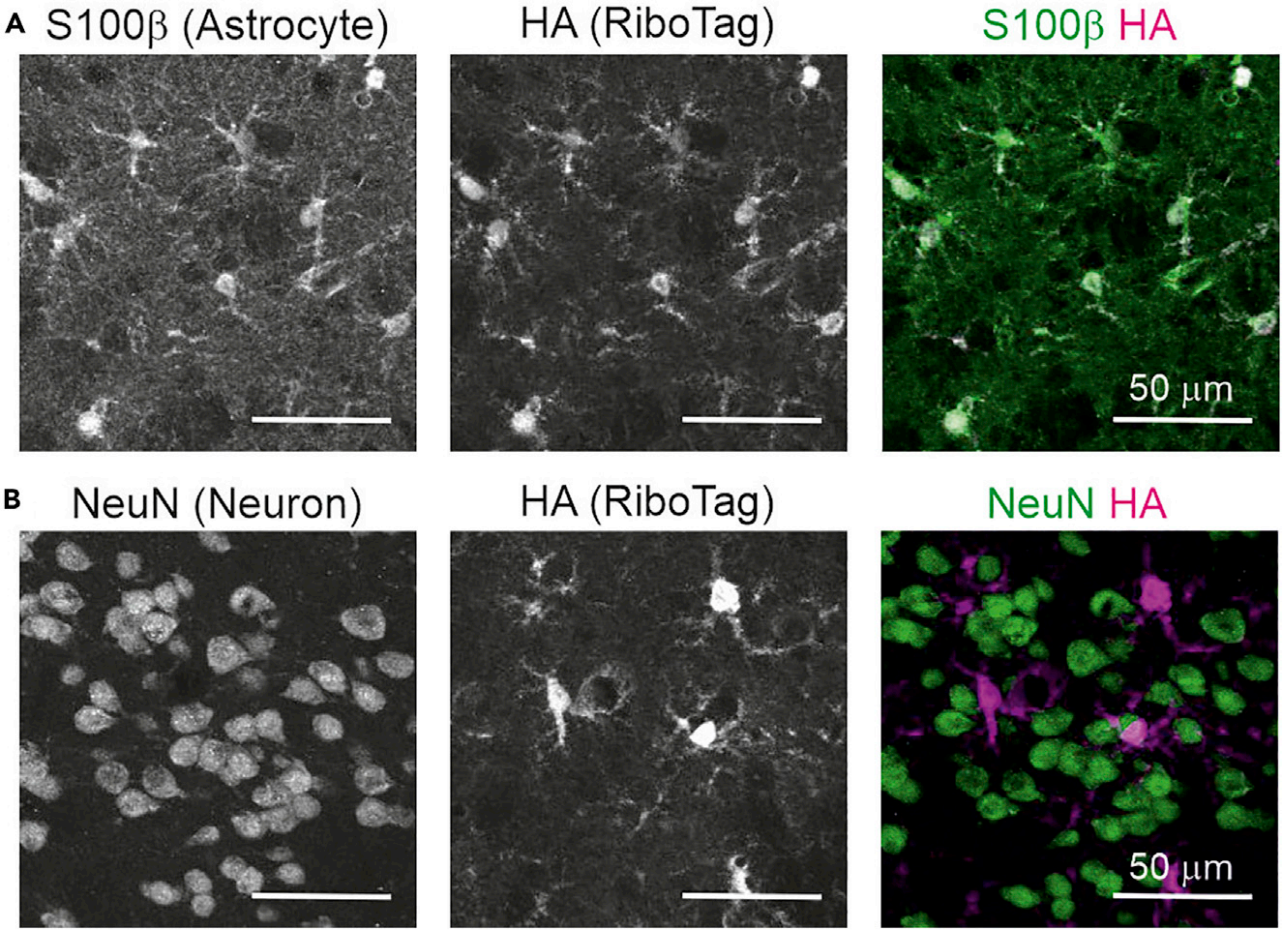
Representative images of RiboTag expression in astrocytes (A and B) Immunohistochemistry to assess the colocalization of RiboTag with the astrocyte marker S100β (A) or the neuronal marker NeuN (B). The scale bar represents 50 μm.

3. We recommend checking the cell specificity of RiboTag expression with immunohistochemistry, especially if using new brain regions or Cre mouse lines to explore cells that have not been tested in this setting before. Colocalization of RiboTag and brain cells can be assessed combining anti-HA and cell specific antibodies (Figure 2). A list of antibodies that we have used before can be found in the key resources table.

## Key resources table

**Table:** 

REAGENT or RESOURCE	SOURCE	IDENTIFIER
**Antibodies**		

Mouse anti-HA (1:1000)	BioLegend	Cat# 901514, RRID: AB_2565336
Rabbit anti-HA (1:1000)	Cell Signaling Technology	Cat# 5017, RRID: AB_10693385
Mouse anti-S100β (1:1000)	Sigma-Aldrich	Cat# S2532, RRID: AB_477499
Rabbit anti-NeuN (1:2000)	Cell signaling	Cat# 12943, RRID: AB_2630395
Rabbit anti-Iba1 (1:1000)	Wako	Cat# 019-19741, RRID: AB_839504
Goat anti-CD13 (1:500 with antigen retrieval)	R&D	Cat# AF2335, RRID: AB_2227288
Rat anti-PECAM1 (1:500)	BD Biosciences	Cat# 550274, RRID: AB_393571
Rabbit anti-ERG (1:1000)	Abcam	Cat# ab92513, RRID: AB_2630401

**Bacterial and virus strains**		

AAV2/5-GfaABC_1_D-RiboTag (Template plasmid under recombinant DNA)	UPenn	N/A

**Chemicals, peptides, and recombinant proteins**		

10% NP-40	Roche	Cat# 11 332 473 001
1 M DTT	Sigma-Aldrich	Cat# 646563
β- Mercaptoethanol	Sigma-Aldrich	Cat# 444203
Protease inhibitor	Sigma-Aldrich	Cat# P8340
RNasin	Promega	Cat# N2115
Cycloheximide	Sigma-Aldrich	Cat# C7698
Heparin	Sigma-Aldrich	Cat# H3393
Pierce A/G magnetic beads	Pierce	Cat# 88803
SuperScript™ IV Reverse Transcriptase	Invitrogen	Cat# 18090010
Ovation PicoSL WTA System V2	NuGen	Cat# 3312
Fast Sybr Green Master Mix	Applied Biosystems	Cat# 4385612
Tris	Sigma-Aldrich	Cat# T3253
KCl	Sigma-Aldrich	Cat# P9333
MgCl_2_	Sigma-Aldrich	Cat# M0250

**Critical commercial assays**		

RNeasy Micro kit	QIAGEN	Cat# 74004

**Experimental models: Organisms/strains**		

B6N.FVB-Tg(Aldh1l1-cre/ERT2)1Khakh/J Mus musculus, hemizygous, 2–3 month old, male and females	JAX	Cat# JAX:031008, RRID: IMSR_JAX:03 1008
B6N.129-Rpl22^tm1.1Psam^/J mice Mus musculus, heterozygous, 2–3 month old, male and females	JAX	Cat# JAX:011029, RRID: IMSR_JAX:01 1029

**Oligonucleotides**		

qRT-PCR primers	Own design; first reported in this manuscript	Table 1

**Recombinant DNA**		

Plasmid to produce AAV2/5-GfaABC_1_D-RiboTag	Yu et al. (2018)	RRID: Addgene_111 811

**Other**		

RNAse/DNAse-free H_2_O	Invitrogen	Cat# 10977-035
Tube rotator	Miltenyi Biotec	Cat# 130-090-753
Magnetic stand	Invitrogen	Cat# 12321D
RNAse/DNAse-free 1.5 mL tubes	Axygen	Cat# MCT-185-C
Filter pipette tips	STARLAB	Cat# S1122-1830
Micropipettes	Eppendorf	Cat# 3123000900
Petri dish for dissection	Brand	Cat# 455742
Dissection microscope	Leica	Cat# S9 E Stereomicroscope
Surgical tools for dissection	F.S.T.	Cat# 11210-20, 11252-11271-30,00,
Dounce homogenizer 2 mL	Kimble	Cat# 885303-002
Pestle A	Kimble	Cat# 885301-002
Pestle B	Kimble	Cat# 885302-002
Tube rotator	Miltenyi Biotec	Cat# 130-090-753,
Magnetic stand	Invitrogen	Cat# CAT12321D
Refrigerated centrifuge	Eppendorf	Cat# 5427R
80°C refrigerator	Phcbi	Cat# MFD-DU702VX-PE

## Materials and equipment

Stock solutions that will be necessary to prepare the homogenization and wash solutions described below can be prepared in advance and stored up to 1 year at 4°C.

• 1.5 M, pH 7.4 Tris in RNase free water.
• 1 M KCl in RNase free water.
• 1 M MgCl_2_ in RNase free water.
• 5 mg/mL Cycloheximide in RNase free water.
• 100 mg/mL Heparin in RNase free water.

**CRITICAL:** Cycloheximide is considered a hazardous chemical according to Regulation (EC) No 1272/2008; wear personal protective equipment when handling. Cycloheximide waste needs to be collected and disposed of according to institute regulations.

Prepare the solutions that will be necessary to perform the protocol. These solutions should be kept at 4°C or on ice.

• Homogenization buffer (HB) – It can be stored at 4°C for two months.

**Table:** 

Reagent	Final concentration	Amount for 10 mL
RNase free H_2_O, 4°C		7,550 μL
1.5 M pH 7.4 Tris, 4°C	50 mM	333 μL
1 M KCl, 4°C	100 mM	1,000 μL
1 M MgCl_2_, 4°C	12 mM	120 μL
10% NP-40, 4°C	1%	1,000 μL

• Supplemented homogenization buffer (S-HB) – It should be made fresh on the day of the experiment and kept at 4°C.

**Table:** 

Reagent	Final concentration	Amount for 5 mL
Homogenization buffer, 4°C		4,702 μL
1 M DTT, room temperature (RT) for up to 2 years	1 mM	5 μL
100× Protease inhibitors, −20°C	1×	50 μL
40 U/μL RNasin, −20°C	0.2 U/μL	25 μL
5 mg/mL Cycloheximide, 4°C	100 μg/mL	100 μL
100 mg/mL Heparin, 4°C for up to 2 years	1 mg/mL	50 μL

**CRITICAL:** DTT is considered a hazardous chemical according to Regulation (EC) No 1272/2008; wear personal protective equipment when handling. DTT waste needs to be collected and disposed of according to institute regulations.

• High salt buffer – It can be prepared fresh on day 1 or at the beginning of day 2 and kept at 4°C.

**Table:** 

Reagent	Final concentration	Amount for 10 mL
RNase free H_2_O, 4°C		5,340 μL
1.5 M pH 7.4 Tris, 4°C	50 mM	333 μL
1 M KCl, 4°C	300 mM	3,000 μL
1 M MgCl_2_, 4°C	12 mM	120 μL
10% NP-40, 4°C	1%	1,000 μL
1 M DTT, 20°C–25°C	1 mM	10 μL
5 mg/mL Cycloheximide, 4°C	100 μg/mL	200 μL

***Note:*** Molarity of pure water calculated with a density of 1 g per mL.

• RNeasy Micro QIAGEN kit lysis buffer – It can be kept at room temperature (RT; 20°C–25°C) for 6 h or at 4°C for up to two days.
  ○ To make RNeasy Micro QIAGEN kit lysis buffer, mix β-mercaptoethanol with RLT QIAGEN buffer in a 1:100 ratio as per manufacturer recommendation (https://www.qiagen.com/gb/resources/resourcedetail?id=e112adfa-cc06-4e29-87f8-4820062ae44e&lang=en). Example for 4 mL: 4 mL of RLT + 40 μL of β- mercaptoethanol.

**CRITICAL:** β-Mercaptoethanol is considered a hazardous chemical according to Regulation (EC) No 1272/2008; wear personal protective equipment when handling. Work under chemical hood. β- Mercaptoethanol waste needs to be collected and disposed of according to institute regulations.

## Step-by-step method details

RNA is highly sensitive to RNases. RNases can be present on your skin and surfaces.

To avoid RNase contamination of your samples and to reduce their activity as much as possible, please adhere to the following tips:

**CRITICAL:** Wash surfaces, pipettes, tube racks, and pipette boxes before starting using Vikron, 1% bleach or RNase Away and do not touch anything without gloves from here on.

**CRITICAL:** Keep everything cold.

**CRITICAL:** Use pipette tips with filters.

**CRITICAL:** Use DNase/RNase free 1.5 mL tubes, water and PBS.

**CRITICAL:** Frequently change gloves to ensure they are clean.

### Tissue dissection and homogenization (day 1)

**Timing: 1–1.5 h**

**Figure 3:**
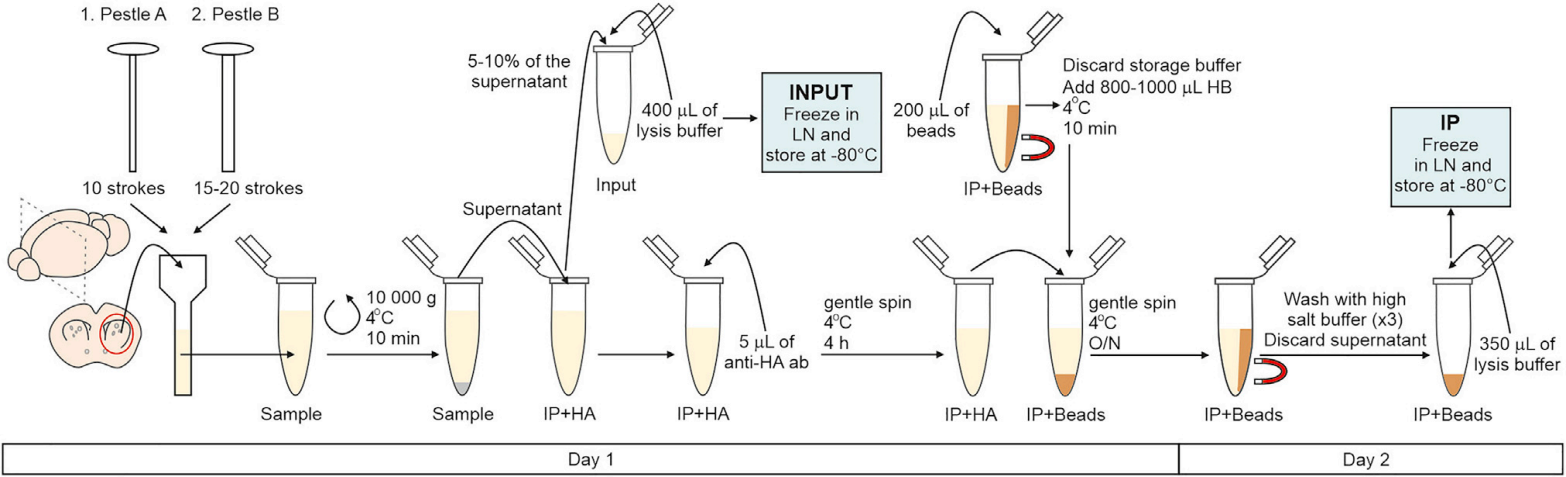
Step-by-step illustration of the RiboTag immunoprecipitation protocol LN – Liquid nitrogen.

**Figure 4:**
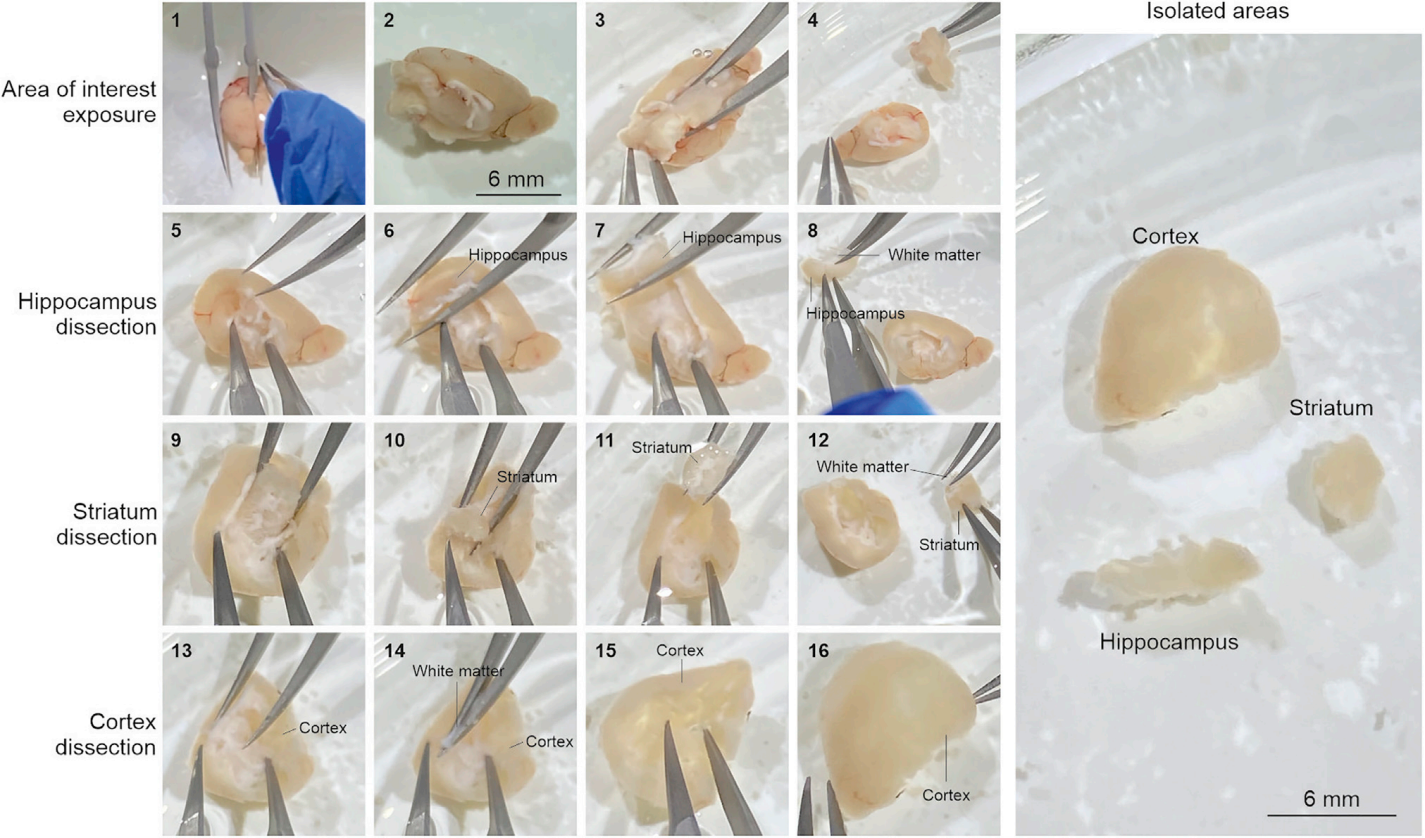
Step-by-step dissection protocol with pictures illustrating how to extract mouse stritatum, hippocampus, and cortex The scale bars represent 6 mm.

In this step, we describe how the brain region of interest, striatum in this case, can be dissected and homogenized (Figures 1C, 3, and 4).

1. Cull and decapitate the mouse following institutionally approved procedures. Extract the brain and put it on a petri dish filled with RNase free PBS under the dissection microscope.
2. Dissect the area of interest. For striatum:
  a. Cut the brain sagittally in two halves (Figure 4).
  b. For each half, remove the thalamus and hippocampus. When the striatum is exposed, scoop the striatum to detach it from the cortex. This can be achieved with fine curved forceps (Figure 4).
  c. Remove the corpus callosum that may have remained bound to the striatum until is clean (Figure 4).
3. Put the two striata from a mouse into the Dounce homogenizer and homogenize the samples at 2%–10% weight/volume, i.e., 1 mL of S-HB solution for two striata from the same brain (30 mg).
  a. Homogenize first with pestle “A” up and down 10 times.
  b. Homogenize then with pestle “B” for 15–20 times.

***Note:*** In between homogenization steps the Douncer can be kept on ice to avoid heating of the sample due to the friction.

***Note:*** Homogenization steps can be repeated if the tissue does not appear to homogenize, as long as the sample stays cold.

4. Transfer the homogenized sample to a 1.5 mL tube labeled with the sample ID.

**CRITICAL:** Between samples, clean the Douncer with RNase free water 3 times (5–10 strokes per pestle each time) to avoid contamination between samples.

**CRITICAL:** During tissue dissection and homogenization, speed is very important. RNAs are exposed to RNases as soon as the mouse is culled. Even though the homogenization buffer contains RNase inhibitors, the probability of RNA degradation increases with time. The homogenized samples should be kept on ice until the entire group is processed. We recommend spending no more than 90 min for the dissection and homogenization step to preserve RNA integrity.

### Sample processing (day 1)

**Timing: 4.5 h**

On day 1, the sample processing involves clearing the homogenized samples from brain debris, collecting a whole tissue (INPUT) sample for RNA extraction, e incubating the rest of the sample with anti-HA antibody and magnetic beads to pull down cell specific RNAs on Day 2 (Figures 1C and 3). 1.5 mL collection tubes for this step can be labeled in advance as IP+HA, INPUT, IP+BEADS, one of each per sample.

5. Centrifuge the samples in 1.5 mL tubes at 4°C/10,000 g/10 min.
6. Transfer supernatant to the tube labeled IP+HA.
7. INPUT SAMPLE.
  a. Take 5%–10% of the supernatant in the tube IP+HA and transfer it to the INPUT sample tube. If the striata were homogenized with 1 mL of S-HB, 50–100 μL can be transferred to the INPUT tube.
    ***Note:*** With 5% of the supernatant for the INPUT sample, we usually obtain 4 times the RNA yield of its corresponding IP sample. If more INPUT RNA is needed, up to 10% of the supernatant can be taken. We do not recommend taking more than 10% since it would decrease significantly the IP RNA amount.
  b. Add 400 μL of Lysis Buffer (1:100 β-mercaptoethanol:RLT buffer from RNeasy kit).
  c. Vortex 10–30 s.
  d. Freeze in liquid nitrogen or dry ice and store at −80°C.
8. IP SAMPLE.
  a. Add 5 μL of anti-HA antibody to each IP sample (in the IP+HA tube), this is a ratio of ∼1:200 vol/vol and 1 μL of antibody for 6 mg of tissue.
    ***Note:*** We typically use mouse anti-HA from BioLegend but rabbit anti-HA from Cell signaling also works (see key resources table).
  b. Incubate for 4 h at 4°C on a gentle spinner at 20 revolutions per minute (rpm; for Miltenyi tube rotator).
  c. Pierce beads preparation (about 30 min before the 4 h IP+HA incubation ends):
    i. Vortex the bead stock vigorously before using to homogenize the beads.
    ii. Add 200 μL of beads to the IP+BEADS 1.5 mL tubes.
    iii. Place the tubes on the magnet and discard the storage buffer solution.
    iv. Add 800–1,000 μL of HB to the beads immediately.
    v. Vortex 10–30 s.
    vi. Incubate the beads at 4°C on a gentle spinner for 10 min at 20 rpm.
  d. Place the IP+BEADS tube on the magnet and discard the buffer.
  e. Quickly add the IP+HA sample to its corresponding IP+BEADS tube.
  f. Incubate 14–16 h at 4°C on a gentle spinner at 20 rpm.
    **CRITICAL:** Do not let the beads dry between washes.
    ***Note:*** Protocol could be optimized for shorter incubation times. However, RNA amount and quality should be checked first.

### Sample processing (day 2)

**Timing: 1 h**

On day 2, the IP samples will be washed several times before mixing them with the lysis buffer for RNA extraction (Figures 1C and 3).

9. Place IP+BEADS tubes on the magnetic rack.
10. Discard the supernatant; the HA-ribosome/RNA complex will be bound to the beads.
11. Wash the beads adding 800 μL of high salt buffer to each sample immediately after removing the liquid.
12. Incubate at 4°C on a gentle spinner for 10 min.
13. Repeat the washes a total of 3 times.
14. Place tubes on the magnetic rack.
15. Discard the high salt buffer.
16. Add 350 μL of Lysis Buffer (1:100 β-mercaptoethanol:RLT buffer from RNeasy kit) to the beads of each sample immediately.
17. Vortex vigorously for 30 s (to break apart the antibody-bead-protein bond).
18. Freeze in liquid nitrogen or dry ice and store at −80°C.

**CRITICAL:** Do not let the beads dry between washes.

### RNA purification

**Timing: 1 h**

RNA purification will be performed following QIAGEN recommendations. It can be done any time after the samples processing steps (Figure 1D). Nonetheless, to ensure RNA integrity, we recommend not to keep the samples longer than 3 months in the −80°C freezer.

19. Thaw the INPUT and IP+BEADS samples on ice.

**CRITICAL:** For the IP RNA, remove the beads (now unbound from the ribosomes and RNA) placing the tubes on the magnetic rack and use the supernatant for RNA purification with RNeasy Micro QIAGEN kit.

20. Proceed with the RNeasy Micro QIAGEN kit following the manufacturer’s instructions (https://www.qiagen.com/gb/resources/resourcedetail?id=e112adfa-cc06-4e29-87f8-4820062ae44e&lang=en).

**CRITICAL:** Purified RNA should be stored at −80°C and freeze/thaw cycles should be avoided to preserve RNA quality and avoid degradation. Before freezing we recommend to set aside an aliquot of the sample for quality assessment, the volume will depend on the assay used (see below). This way the sample that will be used for the translatome assessment does not need to be thawed until the day of the experiment.

### RNA integrity evaluation

**Timing: 1 h**

**Figure 5:**
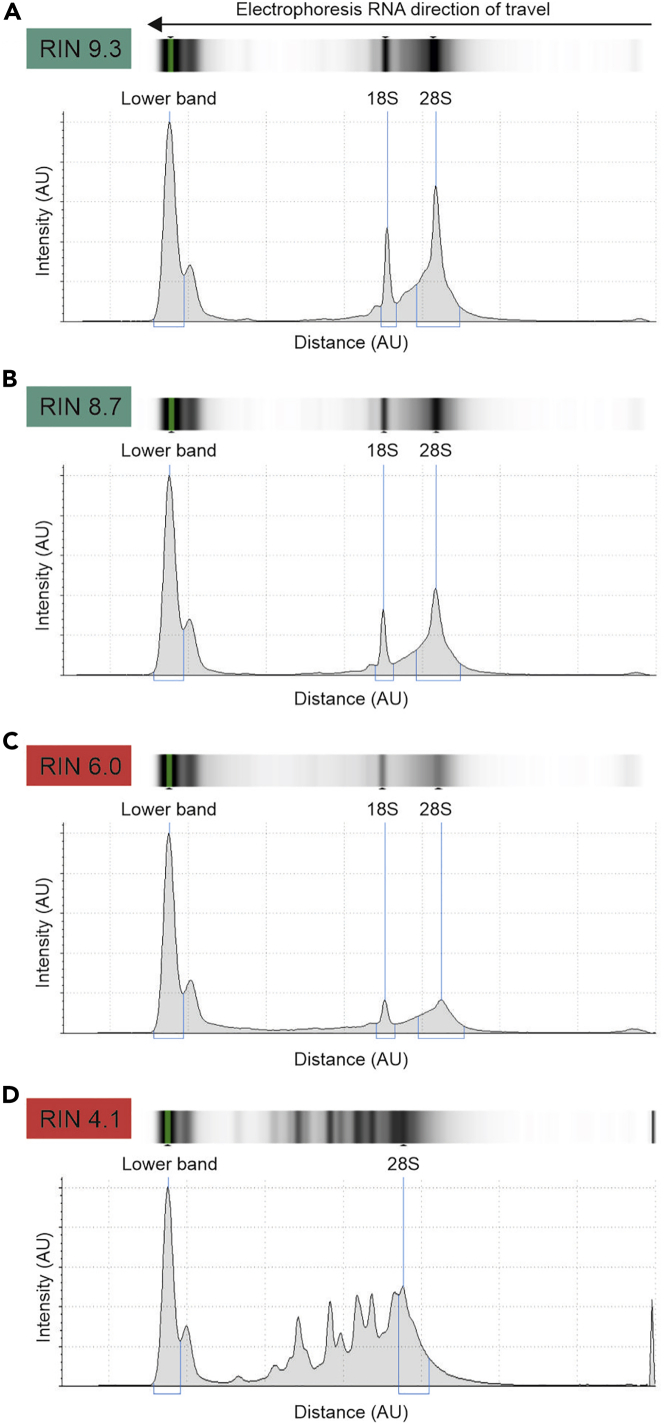
Example of IP RNA integrity assessment by Agilent Bioanalyzer (A and B) Examples of good quality, high RIN RNA with electrophoresis gel picture at the top and electropherogram at the bottom. (C) Example of poor quality, medium RIN RNA with electrophoresis gel picture at the top and electropherogram at the bottom. (D) Example of bad quality, low RIN RNA with electrophoresis gel picture at the top and electropherogram at the bottom. Automatic detection of the lower band, and 18S and 28S ribosomal RNA bands are highlighted with a blue line.

RNA integrity assessment is needed to ensure that RNA has not degraded during the purification protocol (Figures 1D and 5, and Schroeder et al., 2006 for more information). This is important because if the RNA molecules are fragmented, the amplification necessary for quantitative real time polymerase chain reaction (qRT-PCR) or RNA-seq experiments will be affected by the shortened RNA length and consequently the gene expression results will be less accurate.

21. RNA integrity and abundance can be assessed using for example an Agilent Bioanalyzer.

***Note:*** This instrument performs a miniature electrophoresis with 1–2 μL of the RNA sample to determine its integrity based on the electrophoresis band pattern that is analyzed by the software. It provides an RNA integrity number (RIN) and an estimation of the RNA concentration. The RIN scale goes from 0, highly degraded RNA, to 10, RNA with high integrity. RNA samples with RIN > 8 are ideal for qRT-PCR or RNA-seq experiments (Figure 5).

### Cell specificity assessment

**Timing: 1–2 days**

**Figure 6:**
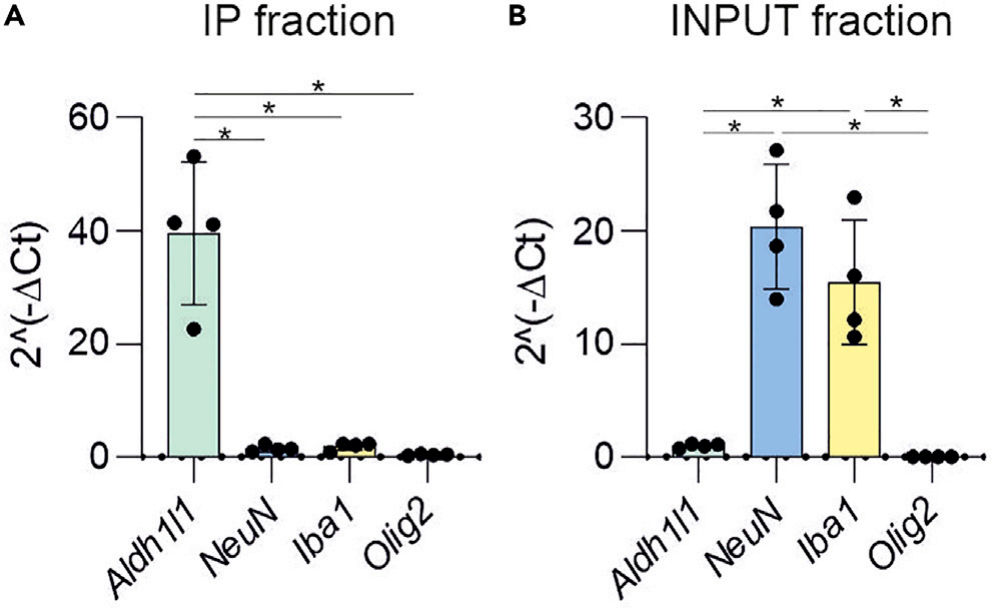
Example of cell specificity assessment by qRT-PCR in *Aldh1l1*-CreERT2::RiboTag brain samples (A) RNA expression as 2^-Δ Ct (*gene of interest*) – Ct (*Rplp0*)^ of different cell markers in the IP fraction shows enrichment of the astrocyte marker *Aldh1l1*. Parametric data. RM one-way ANOVA (p=.008) followed by Tukey’s multiple comparison test (∗p < .05). (B) RNA expression as 2^-Δ Ct (*gene of interest*) – Ct (*Rplp0*)^ of different cell markers in the INPUT fraction shows no enrichment of the astrocyte marker *Aldh1l1* over other cell markers. Nonparametric data. Friedmand test (p=.001) followed by Dunn’s multiple comparison test (∗p < .05). *Aldh1l1*: astrocyte, *NeuN*: neuron, *Iba-1*: microglia, *Olig2*: oligodendrocyte markers. All markers were normalized to the housekeeping ribosome-protein encoding gene *Rplp0*. Data are represented as mean ± SEM. The quantitative data for this figure can be found in Table S1.

We recommend checking the cell specificity of the RNA purified if the protocol is performed for the first time, you are investigating new brain regions, or using new CRE mouse lines (Figure 1D). To check RNA cell specificity, qRT-PCR can be used. To this aim, specific primers targeting RNAs enriched in the cell of interest and primers targeting RNAs enriched in potential contaminating cells should be used (Figure 6, Table S1). Input sample can be used as a control for non-enrichment of cell-type RNAs.

22. Transcribe complementary DNA (cDNA) from the purified RNA using SuperScript™ IV Reverse Transcriptase following manufacturer instructions (https://tools.thermofisher.com/content/sfs/manuals/SSIV_Reverse_Transcriptase_UG.pdf). With this enzyme we reverse transcribe 20 ng of RNA.
  a. Once the cDNA is generated, make a 1:10 dilution of the cDNA stock.
  b. Use 5 μL of the cDNA stock for each qRT-PCR reaction.

***Note:*** If the amount of RNA is not sufficient to complete the qRT-PCR experiment, Ovation PicoSL WTA System V2 cDNA amplification kit can be used for transcription and amplification, instead of the SuperScript transcriptase, following the manufacturer instructions (https://www.tecan.com/hubfs/HubDB/Te-DocList/UG_Ovation_PicoSL_WTA_System_V2.pdf).

23. qRT-PCR can be performed in any qRT-PCR system. To measure the amplification of cDNA during qRT-PCR you can use Fast Sybr Green Master Mix or similar and follow manufacturer protocol (https://tools.thermofisher.com/content/sfs/manuals/cms_046776.pdf).
  a. For the reaction to take place, Fast Sybr Green Master Mix can be mixed with 5 μL of the 1:10 diluted cDNA stock per reaction and a pair of gene specific oligonucleotides (Table 1).

**Table 1:** Oligonucleotide sequences for cell specificity assessment with qRT-PCR

Gene (cell type)	Orientation	Oligonucleotide sequence	Product length
*Adh1l1* (Astrocytes)	Forward	ATGATCATCTCTCGGTTTGCTGA	175
	Reverse	CATCGGTCTTGTTGTATGTGTTG	
*Rbfox3* (Neurons)	Forward	GCGGTCGTGTATCAGGATGG	178
	Reverse	CGATGCTGTAGGTTGCTGTG	
*Pecam 1* (Brain endothelial cells)	Forward	GGTCGACCCTAATCTCATGG	155
	Reverse	AATACGTGCACAGGACTCTCG	
*Olig2* (Oligodendrocytes)	Forward	GAACCCCGAAAGGTGTGGAT	152
	Reverse	GCCCCAGGGATGATCTAAGC	
*Pdgfrb* (Pericytes)	Forward	CTATGCGAGCCTTCCACGAG	185
	Reverse	ACTTTTGAGGTCTCTGCAGGTAG	
*AifI* (Microglia)	Forward	TCCCCCAGCCAAGAAAGCTA	159
	Reverse	GATGTGACCCACTAGGAGCG	
*Rplp0* (Housekeeping)	Forward	CAGGCGTCCTCGTTGGAG	194
	Reverse	ATCTGCTGCATCTGCTTGGAG

***Note:*** The amount of cDNA used for the reaction may vary depending on the level of expression of the gene that will be assessed. If the gene normally has low expression levels, you may need to add more cDNA in order to detect it with qRT-PCR. We recommend performing tests with different dilutions of cDNA before the final experiment, to assess the best amount for your application.

***Alternatives:*** Taqman probes can be used as an alternative to Sybr Green and oligonucleotides.

24. Cell specific gene expression is calculated relative to the housekeeping *Rplp0* based on their Ct values using the formula: 2^-ΔCt (ΔCt = Ct (gene of interest) – Ct (housekeeping gene)^ (Schmittgen and Livak, 2008).

***Note:*** Relative cell enrichment calculations, e.g., expression of an astrocyte gene/neuron gene, can be misleading because they indicate enrichment of a marker over another cell marker but do not show if the level of expression of genes from the non-targeted cell are considerable. Instead, we recommend assessing and presenting the expression of cell-specific genes relative to one or two housekeeping genes. They can also be presented as a ratio of enrichment in the IP vs its corresponding INPUT samples. This cell specificity assessment should not be taken as a substitute for testing with immunohistochemistry, detailed above (Figure 2). Immunohistochemistry of the HA tag and a marker targeting the cell of interest and other unrelated cell types is necessary to assess cell specific RiboTag expression.

25. For the statistical analysis of the qRT-PCR results:
  a. Check if your replicates follow normal distributions with a normality test (e.g., Kolmogorov-Smirnov test).
  b. Check if the variance is homogeneous between conditions with tests that assess homogeneity of variance (e.g., Brown-Forsythe test).
  c. If data are normally distributed and variances are homogeneous between conditions, use repeated measure (RM) one-way ANOVA test.
  d. If not, use Friedman’s test.
  e. If c or d show statistical significance, differences between specific groups can be assessed with post-hoc tests like Tukey’s or Dunn’s respectively.

***Note:*** Since several genes are measured in the same sample it is important to take into account the matching effect between them. Both RM one-way ANOVA and Friedman’s test compare the means of three or more matched groups.

## Expected outcomes

The RNA yields expected from the astrocytes of one mouse striata when using RiboTag::*Aldh1l1-CreERT2* mice is 200–1,000 ng. The yield may increase if the RiboTag gene is in homozygosity. Using the RiboTag AAVs, striatum astrocyte RNA yield is between 500–1,500 ng per mouse.

RNA quality, lack of degradation or fragmentation, is quantified through the RIN. RNA samples should display a RIN > 7.5. As an example, in our latest experiment, 92% of our samples showed a RIN > 7.5 (24 out of 26 samples) (Figure 5).

When the cell enrichment is pure enough, the expression of genes of non-targeted cells is close to 0 and the expression of your cell of interest is at least one order of magnitude higher than the expression of other cell markers (Figure 6).

The number of genes identified by RNA-seq in striatal samples was of 20263 (Chai et al., 2017). We detected 10,000–13,000 genes with an abundance in fragment per kilobase per million (FPKM) > 1.

## Limitations

Our protocol allows the study of cell-specific transcriptomes with RNA-seq at high sequencing depth, i.e., it allows assessment of genes that are not highly expressed and usually missed when performing single-cell (sc)RNA-seq. Moreover, unlike scRNA-seq, cell dissociation is not needed preceding the RNA purification. This has two main advantages, (1) our method captures RNAs that are translated in subcellular compartments such as the astrocyte endfeet that are ripped off with cell isolation protocols, (2) the transcriptome detection is not biased by lower sampling of cells that are more susceptible to dissociation treatments in healthy brain or that become more fragile in disease models. In addition, this method can be used to investigate the translatome of cells that are traditionally challenging to study through scRNA-seq due to the difficulty of obtaining enough cells of that type, e.g., pericytes or stem cells. However, this protocol has some limitations to consider. RiboTag only allows the cell-specific translatome assessment of one cell type per mouse. The variety of cell types that you can assess is dependent on the availability of Cre mouse lines or AAV promoters. If Cre mice need to be used, breeding schemes can be time and resource consuming. In addition, the description of intraregional cell subpopulations may be missed. Nonetheless, to gain understanding on cell subpopulations, our protocol can be combined with immunohistochemistry or RNA-scope; this has provided insightful information about astrocyte subpopulations (Chai et al., 2017; Díaz-Castro et al., 2019). The development of new genetic tools will permit the targeting of newly identified cell subpopulations that can be assessed with the method presented here. Finally, it is important to highlight that this protocol is based on the immunoprecipitation of polyribosomes. Therefore, it only allows the detection of ribosome-associated RNAs, e.g., messenger RNAs, ribosomal RNAs, regulatory microRNA, etc. This could be a limitation or an advantage depending on the biological question.

## Troubleshooting

### Problem 1

RiboTag only expresses in a fraction of the cells you aim to target (“before you begin” section).

### Potential solution

The spread of expression of RiboTag should be assessed in advance with immunohistochemistry, e.g., measuring RiboTag-HA expression (with rabbit anti-HA Cat# 5017, RRID: AB_10693385) in S100β positive astrocytes (with mouse anti-S100β Cat# S2532, RRID: AB_477499). If it appears that not as many cells as expected with your Cre line express RiboTag, tamoxifen dose could be increased to ensure wide expression of RiboTag in your cell of interest. This will avoid an unintentional sampling of transcriptomes of certain cell subpopulations.

### Problem 2

AAV mediated RiboTag expression appears in non-desired cells (“before you begin” and “cell specificity assessment” sections, step 24).

### Potential solution

Excessive AAV injection could lead to expression of RiboTag in non-desired cell types. AAV dose may need to be reduced.

### Problem 3

Highly variable RNA yield (“RNA integrity evaluation” section, step 21).

### Potential solution

Variability in the RNA yield can have several causes, hence several solutions:

• If the tissue dissection is not consistent between mice, the RNA yield could vary a lot between samples. We recommend practicing the dissection of the brain region of interest until it becomes a routine and consistent. To assess uniformity among dissections the tissue can be weighed. In addition, whole tissue RNA can be extracted and quantified with a nanodrop to ensure there is not too much variation.
• If Cre/ERT2 mice are used to activate RiboTag expression, it is possible that the tamoxifen administration is not consistent and in every mouse the number of cells expressing RiboTag varies too much. This could happen, for example if the intraperitoneal injections are not administered appropriately. Tamoxifen is diluted in oil and it will be very dense, injections need to be slow and steady. The consistency of RiboTag activation should be assessed with immunohistochemistry and by counting RiboTag expressing cells across at least 4–6 animals.
• If RiboTag AAV is used, variabilities in RNA yield could be due to two reasons:
  ○ Coordinates of intracranial injections are not consistent between mice. If this happens, when the tissue is dissected, the amount of the dissected tissue that expresses RiboTag can be very different. This can be more challenging with small brain areas. To avoid this from happening, please make sure the stereotaxic frame is properly calibrated and that the mouse head is hold steady without movement.
  ○ The volume of virus injected is different between mouse models. This can happen if there are clogs or bubbles in your AAV injection needle or tubbing. Please check carefully the injection system to make sure is clean and with no air.

To ensure AAV injections are uniform and always target the same regions, immunohistochemistry of several slices along bregma should be performed to assess RiboTag expression position and spread in at least 4–6 mice.

### Problem 4

Low RIN RNA (“RNA integrity evaluation” section, step 21).

### Potential solution

If using Agilent tapestation to calculate RIN numbers, low RIN can be observed for two main reasons:

• The gel electrophoresis tape is not the adequate for the RNA concentration range you are trying to assess. We recommend performing a pilot RNA concentration assessment with nanodrop to get a rough idea of the RNA concentration of your sample and chose the gel tape and buffer that is designed for your concentration range.
• The gel tape is expired. Please follow the manufacturers guidance on the tape lifetime. Gel tapes can be reused if not all lanes are filled on a single experiment, but once the gel tape is taken out of its original package it will need to be used within a month.

Low RIN RNA can also be due to RNA degradation. If this is the case, please double check that you have followed all the precautions listed on the protocol to avoid RNase contamination and activity. Make sure that the RNasin buffer (RNAse inhibitor) is not expired or have been left at RT.

### Problem 5

Low RNA yield (“RNA integrity evaluation” section, step 21).

### Potential solution

There are several solutions to low RNA yields.

• If using the RiboTag mouse line, maintain the RiboTag gene in homozygosity. We observe higher yields in samples from homozygous mice.
• Tissue from several mice can be pulled and homogenized together when the brain region of interest is small (Díaz-Castro et al., 2021).
• RNA amplification kits for picogram amounts of starting RNA are available (e.g., Ovation PicoSL WTA System V2) to be able to perform RNA-seq even when the RNA yield is very small.

## Resource availability

### Lead contact

Further information and requests for resources and reagents should be directed to and will be fulfilled by the lead contact, Blanca Díaz-Castro (b.diaz-castro@ed.ac.uk).

### Materials availability

This study did not generate new unique reagents. However, the plasmid used to generate astrocyte RiboTag AAVs can be found on Addgene (RRID: Addgene_111811) and the RiboTag and astrocyte specific Cre mouse lines at Jax Cat# JAX:011029, RRID: IMSR_JAX:011029 and Cat# JAX:031008, RRID: IMSR_JAX:031008 respectively.

## Data Availability

In this study we generated qRT-PCR data and analyses. The published article includes all datasets generated or analyzed during this study. This study did not generate codes or analyzed datasets.

## Supplemental information

Table S1. qRT-PCR individual-sample raw data, related to Figure 6 and step 24
